# Impact of psychosocial factors on cardiovascular morbimortality: a prospective cohort study

**DOI:** 10.1186/1471-2261-14-135

**Published:** 2014-10-03

**Authors:** Cília Mejía-Lancheros, Ramón Estruch, Miguel-Angel Martínez-González, Jordi Salas-Salvadó, Olga Castañer, Dolores Corella, Fernando Arós, Enrique Gómez-Gracia, Miquel Fiol, José Lapetra, Lluís Serra-Majem, Xavier Pintó, Emilio Ros, Javier Díez-Espino, Josep Basora, José-V Sorlí, Rosa-Maria Lamuela-Raventos, Valentina Ruiz-Gutiérrez, Miguel-Ángel Muñoz, on behalf of the PREDIMED Study Investigators

**Affiliations:** Department of Paediatrics, Obstetrics, Gynaecology and Preventive Medicine, Universitat Autònoma de Barcelona, Bellaterra, Spain; CIBER Fisiopatología de la Obesidad y Nutrición (CIBERobn), Instituto de Salud Carlos III, Madrid, Spain; PREDIMED (Prevención con Dieta Mediterránea) Network (RD 06/0045), Instituto de Salud Carlos III, Madrid, Spain; Department of Internal Medicine of Hospital Clinic, IDIBAPS, University of Barcelona, Barcelona, Spain; Preventive Medicine and Public Health, University of Navarra, Pamplona, Spain; Human Nutrition Department, Hospital Universitari Sant Joan, Institut d’Investigació Sanitaria Pere Virgili, Universitat Rovira i Virgili, Reus, Spain; Cardiovascular Risk and Nutrition Research Group of Institute Hospital del Mar (IMIM), Barcelona, Spain; Department of Preventive Medicine, University of Valencia, Valencia, Spain; Department of Cardiology, University Hospital of Alava, Vitoria, Spain; Department of Preventive Medicine, University of Malaga, Malaga, Spain; Institute of Health Sciences (IUNICS), University of Balearic Islands, and Hospital Son Espases, Palma de Mallorca, Spain; Department of Family Medicine, Primary Care Division of Seville, San Pablo Health Center, Seville, Spain; Department of Clinical Sciences, University of Las Palmas de Gran Canaria, Las Palmas, Spain; Lipids and Vascular Risk Unit, Internal Medicine, Hospital Universitario de Bellvitge, Hospitalet de Llobregat, Barcelona, Spain; Lipid Clinic, Department of Endocrinology and Nutrition of Hospital Clinic, Institut d’Investigacions Biomèdiques August Pi I Sunyer, University of Barcelona, Barcelona, Spain; Centro de Salud de Tafalla, Servicio Navarro de Salud, University of Navarra, Pamplona, Spain; Primary Care Division, Institut Català de la Salut, Institut d’Investigació en Atenció Primària IDIAP Jordi Gol, Tarragona-Reus, Spain; Primary Care Division, Valencia Institute of Health, Valencia, Spain; Department of Nutrition and Food Science, School of Pharmacy, Xarxa de Referència en Tecnologia dels Aliments, Instituto de Investigación en Nutrición y Seguridad Alimentaria, University of Barcelona, Barcelona, Spain; Instituto de la Grasa, Consejo Superior de Investigaciones Científicas, Sevilla, Spain; Institut Català de la Salut, Barcelona, Spain; Institut d’Investigació en Atenció Primària IDIAP-Jordi Gol, Barcelona, Spain

**Keywords:** Stroke, Acute myocardial infarction, Cardiovascular death, Educational level, Socioeconomic position, Depression, Social support, Health inequalities

## Abstract

**Background:**

Whilst it is well known that psychosocial determinants may contribute to cardiovascular diseases (CVD), data from specific groups are scarce. The present study aims to determine the contribution of psychosocial determinants in increasing the risk of cardiovascular events (myocardial infarction and stroke), and death from CVD, in a high risk adult population.

**Methods:**

Longitudinal prospective study of 7263 patients (57.5% women), mean age 67.0 (SD 6.2) free from CVD but at high risk, with a median follow-up of 4.8 years (from October 2003 to December 2010). The Hazard Ratios (HRs) of cardiovascular events (myocardial infarction, stroke, and death from cardiovascular causes) related to educational attainment, diagnosed depression (based on medical records), and low social support (number of people living in the household) were estimated by multivariate Cox regression models.

**Results:**

Stroke incidence was associated with low educational level in the whole population (HR: 1.83, 95% CI: 1.09–3.09), and especially in men (HR: 2.11, 95% CI 1.09–4.06). Myocardial infarction and CVD mortality were not associated with any of the psychosocial factors considered.

**Conclusion:**

Adults with low educational level had a higher risk of stroke. Depression and low social support were not associated with CVD incidence.

**Trial registration:**

Clinical trial registration information unique identifier: ISRCTN35739639.

## Background

Cardiovascular disease (CVD) remains the leading cause of mortality worldwide, and in most developed countries is the major origin of disability among elderly people. In 2008, heart attacks and strokes were responsible for 7.3 and 6.2 million deaths, respectively [1]. Its incidence has been strongly related with classic risk factors (hypertension, dyslipidemia, and type 2 diabetes), and poor lifestyles (smoking, physical inactivity, and unhealthy diet) [2, 3]. In recent decades, inadequate psychosocial and living conditions have also been found to be linked to CVD [4, 5]. Individual conditions such as low socioeconomic status, weak social support, depression, and residing in disadvantaged neighborhoods may contribute to socioeconomic inequalities in cardiovascular health [4–9]. Their roles, however, are not yet entirely clear [8, 9] and, in some contexts, not taken into consideration. In Spain, where socioeconomic health disparity is not as pronounced as in some other European countries [10], and CVD incidence is one of the lowest in the world [1, 11, 12], there are few studies which have evaluated the effect of psycho-social factors [13].

### Study aims

The present study aimed at determining whether adverse psychosocial conditions such as lower educational level, depression, and weak social support contribute to increasing the risk of cardiovascular events (myocardial infarction and stroke) and death from CVD in an adult population at high cardiovascular risk.

## Methods

### Study design and population

This is a longitudinal, prospective study embedded within the PREDIMED Study (Prevention with Mediterranean diet) carried out from October 2003 to December 2010, in Spain. Details of PREDIMED study enrollment, design, population, methods, and main results have been described elsewhere [14]. For the purpose of this article, we analyzed 7263 participants (women and men) aged 55–80 years old, at high cardiovascular risk, but free from cardiovascular disease at baseline (97.5% of PREDIMED participants), with complete, available data concerning psychosocial risk factors.

#### Inclusion criteria

participants had to have at least one of the following two conditions: a) Medical diagnosis of type 2 diabetes or receiving insulin or oral hypoglycemic drugs; or having fasting glucose >126 mg/dl or presented casual glucose >200 mg/dl with polyuria, polydipsia, or unexplained weight loss; or glucose > 200 mg/dl in two measurements after an oral glucose tolerance test. b) At least three of the following risk factors: smoking (>1 cig/day during the last month); hypertension (systolic blood pressure > =140 mm Hg or diastolic blood pressure > =90 mmHg or under antihypertensive medication); elevated low-density lipoprotein cholesterol levels (> = 160 mg/dl); low high-density lipoprotein cholesterol levels (<= 40 mg/dl); overweight (body mass index > =25 kg/m^2^); or a family history of premature coronary heart disease (CHD) (definite myocardial infarction or sudden death before 55 years in father or male 1st-degree relative, or before 65 years in mother or female 1st-degree relative). If the HDL-cholesterol level was > =60 mg/dL, one risk factor was subtracted.

#### Exclusion criteria

Participants with any of the following were excluded: documented history of previous cardiovascular disease or severe medical conditions (digestive disease with fat intolerance, advanced malignancy, major neurological, psychiatric or endocrine disease); immunodeficiency; illegal drug use; problematic alcohol intake (chronic alcoholism or total daily alcohol intake >80 g/d); body mass index > 40 kg/m^2^; difficulties or major inconvenience to change dietary habits; impossibility of following a Mediterranean-type diet or understanding the recommendations of the protocol; and lack of autonomy.

Data were collected from medical records, clinical evaluations, and face to face interviews. Validated questionnaires were administered in order to obtain information on nutritional habits [15, 16]. Blood samples for laboratory tests were gathered. All participants gave written informed consent. The study was approved by the Institutional Review Board of Hospital Clinic (Barcelona, Spain), and registered in the Current Controlled Trials (number: ISRCTN35739639, http://www.controlled-trials.com/ISRCTN35739639).

### Study variables

#### Psychosocial conditions

*Educational level*: Educational level was used as a proxy of socioeconomic position (SP) since it is considered a strong determinant of social status that may condition employment and income opportunities [17]. For the purpose of the study, educational attainment in the bivariate analysis was classified into three groups: high education (secondary or university studies), primary education (completed primary school), and less than primary education. In the multivariate analysis, educational level was divided into two groups: high education (secondary or university studies) and low education (up to primary school).

*Social support*: Social support in the household was considered to be low when participants were living alone. Living alone has been reported as a valid proxy to evaluate social support, and has been found to be an independent social risk factor for adverse cardiovascular event outcome [18].

*Depression*: Information regarding history of diagnosis of depression was self-reported by participants during a face to face interview at the inclusion visit and further confirmed in clinical records. In Spain, the diagnosis of depression is carried out by both psychiatrists and GPs following the American Psychological Association clinical criteria (DSM-IV) or other mental health scales (e.g. the Beck Depression Inventory).

*Other co-variables*: socio-demographic variables: Age (years) and gender. Smoking status: Never smoked, former smoker, and current smoker. Alcohol intake: High alcohol consumption (Alcohol consumption more than 20 gr. daily in men and 10 gr. daily in women). Body mass index (BMI): Expressed as weight in kilograms divided by height in meters squared (Kg/m^2^). Cardiovascular risk factors: Hypertension (Medical diagnosis of hypertension or receiving at least one of the following antihypertensive drugs: angiotensin-converting-enzyme inhibitor (ACE inhibitors), diuretics, calcium channel blockers, angiotensin II receptor antagonists, ß-blockers, α-blockers, or other antihypertensive drugs); Diabetes (medical diagnosis of Type 2 diabetes or receiving insulin and/or oral hypoglycemic drugs); Dyslipidemia (medical diagnosis of dyslipidemia or receiving lipid lowering therapy); Family history of premature coronary heart disease (CHD). Mediterranean dietary intervention: (i) Low fat Mediterranean diet (control diet): dietary recommendation which highlighted the consumption of lean meats, low-fat dairy products, cereals, potatoes, pasta, rice, fruit and vegetables. The use of olive oil for cooking and dressing and consumption of nuts, fatty meats, sausages, and fatty fish were discouraged. (ii) Mediterranean diet enriched with extra-virgin olive oil (EVOO): positive dietary recommendation about the Mediterranean diet pattern emphasizing the abundant use of olive oil for cooking and dressing dishes. One liter per week of EVOO was supplied to the participants in this group. (iii) Mediterranean diet enriched with mixed nuts: positive dietary recommendation about the Mediterranean diet pattern with the addition of nuts. Participants received 30 g of mixed nuts per day (15 g of walnuts, 7.5 g of almonds and 7.5 g hazelnuts).

The PREDIMED study dietitians supervised the dietary intervention. Further details about PREDIMED study dietary intervention has been previously published [14].

### End points

#### Composite primary cardiovascular event

The primary end point was a composite variable made up of the first occurrence of cardiovascular death, or myocardial infarction, or stroke (combining ischemic and hemorrhagic). Diagnostic criteria are available in a supplementary appendix of a previously published article [14].

The three cardiovascular events were also considered separately in the analysis in order to individually ascertain the impact of psychosocial factors.

Cardiovascular events were reviewed and confirmed by an Adjudication Committee of the PREDIMED Study consisting of a team of cardiologists, endocrinologists, neurologists, and ophthalmologists. Members of the committee were blinded to the intervention and dietary habits of the participants; for an event to be accepted a consensus was required. Events that had occurred between October 1st, 2003 and December 1st, 2010 were analyzed. Transient Ischemic Attack was not considered as a stroke. All end points were ascertained by regular contacts with participants and/or families, annual revisions of medical records, data from GPs, and consultation of the National Death Index (Spain).

### Statistical analyses

Bivariate analysis was performed using chi-square test for analyzing differences among proportions, and t-student or one-way ANOVA for differences among means. Crude Rate/1000 person-year (95% confidence interval: CI) of cardiovascular events was calculated. Crude and adjusted Hazard ratios (HR) with 95% CI were performed by Cox regression models for the analysis of time-dependent cardiovascular events in relation to psychosocial factors. Multivariate Cox models were adjusted for age, gender, smoking, alcohol consumption, BMI hypertension, type 2 diabetes, dyslipidemia, family history of premature CVD at baseline, and the randomized arm of the Mediterranean dietary intervention during the trial. To assess whether cardiovascular events could be predicted by unfavorable psychosocial factors related to gender, all multivariate adjusted analysis were stratified by sex. The proportional HR assumption from the multivariate Cox models was validated with time-varying tests (Schoenfeld residuals approach) [19]. The global test p-values were ≥ 0.05.

Participants were considered censored when no event was registered during the study or data had been lost during follow-up. Censoring was assumed to be independent of the main study variables and non-informative. The resulting bias was, however, minimized by our close participant follow-up.

On the other hand our study was right censoring because some events had not occurred when the study ended.

Individuals lacking information concerning their psychosocial risk factors were not included. As this represented an insubstantial percentage (2.5%), their exclusion was not expected to greatly affect the internal and external validity of the observed results.

## Results

### Main baseline characteristics of study participants

The mean age of participants was 67.0 years (SD 6.2), and 57.5% were women. Regarding psychosocial factors, 74.8% of the participants had only attained primary education and 2.5% had less than primary school, 18.0% had a previous diagnostic of depression, and 10.0% weak social support (living alone in the household). With respect to cardiovascular risk factors, 14.0% (n = 1020) of the participants were currently smokers, 91.1% (n = 6620) had hypertension, 72.1% (n = 5238) diabetes, 77.8% (n = 5648) dyslipidemia, and 22.2% (n = 1615) reported family history of coronary heart disease.

### Characteristics of the population regarding psychosocial factors

Participants with primary and less than primary education were more frequently older, women, and overweight. They had low social support and a higher percentage of type 2 diabetes and depression. In contrast, they consumed less alcohol and were less frequently smokers. Participants with weak social support were more commonly older, women, with dyslipidemia, depression and low education, but they were less frequently smokers. Depression was associated with women, overweight, dyslipidemia, family history of coronary heart disease, weak social support, and a low educational level, however, they consumed less alcohol and tobacco (Table 1).

**Table 1:** **Characteristics of participants according to psychosocial factors at baseline**

	Education level				Social support in the household			Diagnosis of depression		
	High education*	Primary education	Less than primary education		Live with other	Live alone		None	Yes	
	(N = 1646)	(N = 5433)	(N = 184)		(N = 6535)	(N = 728)		(N = 5954)	(N = 1309)	
	(%)	(%)	(%)	p-value	(%)	(%)	p-value	(%)	(%)	p-value
Age (mean; SD^†^)	64.6 (6.2)	67.7 (6.1)	69.4 (5.0)	<0.001	66.6 (6.1)	70.1 (6.2)	<0.001	67.0 (6.3)	66.8 (6.0)	0.217
Sex (Women)	37.4	62.6	89.7	<0.001	54.8	82.1	<0.001	52.7	79.8	0.001
Type of Mediterranean diet										
Low fat diet	29.0	34.0	26.1	0.453	32.4	34.9	0.130	32.2	34.4	0.096
With extra-Virgin Olive oil	33.8	34.2	42.4		34.7	31.0		34.2	35.1	
With mixed nuts	37.2	31.8	31.0		32.9	34.1		33.6	30.6	
High alcohol consumption^‡^	30.0	18.8	8.2	<0.001	21.5	17.1	0.007	22.5	14.6	<0.001
Smoking status										
Never smoked	41.4	66.5	84.8	<0.001	59.9	73.9	<0.001	58.4	74.5	<0.001
Former smoker	36.9	21.5	9.2		25.5	17.0		27.1	13.5	
Current-smoker	21.7	12.0	6.8		14.6	9.1		14.5	12.0	
Body-mass index (Kg/m^2^)	29.3 (3.7)	30.1 (3.8)	30.7 (4.1)	<0.001	30.0 (3.8)	30.0 (4.2)	0.743	29.9 (3.8)	30.5 (4.0)	<0.001
Cardiovascular risk factors^§^										
Hypertension	90.5	91.2	94.6	0.159	91.0	92.6	0.151	91.1	91.4	0.756
Type 2 diabetes	67.7	73.2	81.0	<0.001	72.1	72.1	0.998	72.1	72.3	0.840
Dyslipidemia	77.5	77.8	79.9	0.764	77.4	81.2	0.019	77.2	80.5	0.008
Family history of premature CHD^¶^	23.7	21.9	20.1	0.231	22.4	21.0	0.404	21.6	25.1	0.007
Living alone	8.3	10.5	12.5	0.022	----	-------	------	9.0	14.7	<0.001
Diagnostic of depression	15.2	18.6	27.7	<0.001	17.1	26.5	<0.001	------	-----	----
Educational level										
High education*	---	-----	----		23.1	18.8	0.022	23.4	19.1	<0.001
Primary education	---	-----	----		74.4	78.0		74.3	77.0	
Less than primary education	---	-----	----		2.5	3.2		2.2	3.9	

*High education means university studies or secondary school.

^†^Standard deviation.

^‡^Alcohol consumption presented 68 missing values in its denominator. Standard deviation.

^§^Hypertension (Medical diagnosis of hypertension or taking antihypertensive treatment), Type 2 diabetes (Medical diagnosis of diabetes or taking antidiabetic treatment) and Dyslipidemia (Medical diagnosis of dyslipidemia or taking lowering-lipid therapy).

^¶^CHD denotes coronary heart disease.

**Figure 1:**
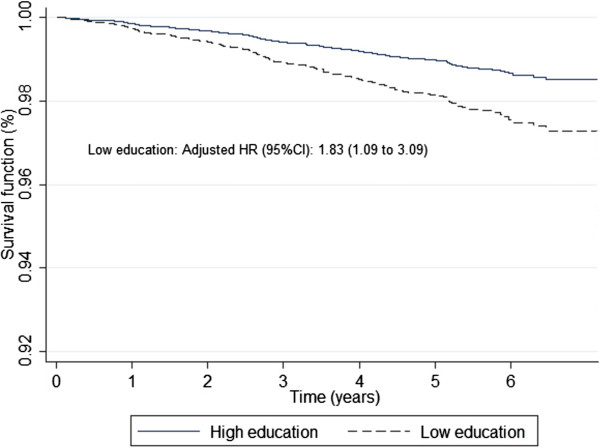
**Survival function of stroke adjusted according education level based on Cox proportional hazard model.** The Cox model was adjusted by Sex, age, smoking, alcohol consumption, body mass index, hypertension, type 2 diabetes, dyslipidemia, family history of premature cardiovascular disease, and type of Mediterranean diet intervention. High education means university education or secondary school, and low education denotes primary education.

The median participant follow-up was 4.8 years (interquartile range 2.8 to 5.8). In our sample 280 CVD events occurred. Participants with a high educational level had 56 events (7.9 per 1000 person-year) versus 224 (9.2 per 1000 person-year) in those with a low educational one. Participants with higher social support had 257 CVD events (9.1 per 1000 person-year) versus 23 (7.5 per 1000 person-year) in those living alone. A total of 248 (9.6 per 1000 person-year) CVD events occurred in people without depression versus 32 (5.7 per 1000 person-year) in those with depression (Table 2). Low educational level was associated with an increased risk of stroke (adjusted Hazard Ratio (HR): 1.83, 95% CI: 1.09–3.09) (Table 2, Figure 1). The risk of stroke was higher in men (Adjusted HR: 2.11, 95% CI: 1.09–4.06) than in women (adjusted HR: 1.46, 95% CI: 0.62–3.43) (Table 3).

**Table 2:** **Incidence and adjusted hazard ratios for cardiovascular events according to psychosocial factors**

	Educational level*		Social support in the household		Diagnosis of depression	
	High education	Low education	Live with other	Live alone	No	Yes
	(N = 1646)	(N = 5617)	(N = 6535)	(N = 728)	(N = 5954)	(N = 1309)
Person-year of follow-up	7069,0	24280,89	28287,36	3062,56	25751,12	5598,79
**Composite primary cardiovascular event**						
Number of events	56	224	257	23	248	32
Crude Rate/1000 person-year (95% CI)	7.9 (5.8–10.0)	9.2 (8.0–10.4)	9.1 (8.0–10.2)	7.5 (4.4–10.6)	9.6 (8.4–10.8)	5.7 (3.7–7.7)
Adjusted Hazard Ratio (95% CI)^†‡^	1.00 (ref)	1.16 (0.85–1.57)	1.00 (ref)	0.85 (0.54–1.32)	1.00 (ref)	0.76 (0.52–1.11)
p-value		0.351		0.458		0.155
**Myocardial Infarction**						
Number of events	24	79	95	8	90	13
Crude Rate/1000 person-year (95% CI)	3.4 (2.0–4.8)	3.3 (2.5–4.0)	3.4 (2.7–4.0)	2.6 (0.8–4.4)	3.5 (2.8–4.2)	2.3 (1.1–3.6)
Adjusted Hazard Ratio (95% CI)^†‡^	1.00 (ref)	1.10 (0.69–1.78)	1.00 (ref)	1.01 (0.48–2.12)	1.00 (ref)	0.89 (0.49–1.62)
p-value		0.686		0.980		0.699
**Stroke**						
Number of events	17	119	127	9	121	15
Crude Rate/1000 person-year (95% CI)	2.4 (1.3–3.5)	4.9 (4.0–5.8)	4.5 (3.7–5.3)	2.9 (1.0–4.9)	4.7 (3.9–5.5)	2.7 (1.3–4.0)
Adjusted Hazard Ratio (95% CI)^†‡^	1.00 (ref)	1.83 (1.09–3.09)	1.00 (ref)	0.56 (0.28–1.12)	1.00 (ref)	0.66 (0.38–1.15)
p-value		0.023		0.102		0.145
**Cardiovascular Death**						
Number of events	25	58	73	10	73	10
Crude Rate/1000 person-year (95% CI)	3.5 (2.2–4.9)	2.4 (1.8–3.0)	2.6 (2.0–3.2)	3.3 (1.2–5.3)	2.8 (2.2–3.5)	1.8 (0.7–2.9)
Adjusted Hazard Ratio (95% CI)^†‡^	1.00 (ref)	0.63 (0.38–1.03)	1.00 (ref)	1.21 (0.60–2.46)	1.00 (ref)	0.93 (0.47–1.84)
p-value		0.064		0.598		0.832

*High education means university studies or secondary school; Low education: up to primary studies.

^†^Multivariable models were adjusted by: age, gender, smoking, alcohol consumption, body-mass index, hypertension, type 2 diabetes, dyslipidemia and family history of premature coronary heart disease, and type Mediterranean diet intervention.

^‡^Test of proportional-hazard assumption (p-value based on the scaled Schoenfeld residuals): Composite primary cardiovascular event model (specific p-value for Educational level: 0.529, Social support: 0.765 and Depression: 0.877); Myocardial infarction model (0.611, 0.717 and 0.914); Stroke model (p-values = 0.204, 0.598 and 0.411); Cardiovascular death model (p-values = 0.810, 0.831 and 0.129).

**Table 3:** **Adjusted hazard ratios for cardiovascular events according to psychosocial factors stratified by gender**

	Composite primary cardiovascular event		Myocardial infarction		Stroke		Cardiovascular death	
	Adjusted HR (95% CI) ^†‡^	p-value	Adjusted HR (95% CI) ^†‡^	p-value	Adjusted HR (95% CI) ^†‡^	p-value	Adjusted HR (95% CI) ^†‡^	p-value
**Men**								
Educational level*								
High education	1.00 (ref)		1.00 (ref)		1.00 (ref)		1.00 (ref)	
Low education	1.25 (0.87–1.80)	0.223	1.34 (0.76–2.35)	0.308	2.11 (1.09–4.06)	0.026	0.65 (0.36–1.16)	0.148
Social support in the household								
Live with others	1.00 (ref)		1.00 (ref)		1.00 (ref)		1.00 (ref)	
Live alone	0.74 (0.30–1.61)	0.512	0.74 (0.18–3.05)	0.682	0.33 (0.46–2.39)	0.272	1.53 (0.47–4.98)	0.477
Diagnosis of Depression								
No	1.00 (ref)		1.00 (ref)		1.00 (ref)		1.00 (ref)	
Yes	0.76 (0.40–1.46)	0.414	0.50 (0.16–1.60)	0.241	0.56 (0.17–1.79)	0.327	1.42 (0.56–3.62)	0.460
**Women**								
Educational level*								
High education	1.00 (ref)		1.00 (ref)		1.00 (ref)		1.00 (ref)	
Low education	0.97 (0.56–1.70)	0.922	0.63 (0.27–1.50)	0.295	1.46 (0.62–3.43)	0.383	0.56 (0.22–1.39)	0.211
Social support in the household								
Live with others	1.00 (ref)		1.00 (ref)		1.00 (ref)		1.00 (ref)	
Live alone	0.81 (0.48–1.37)	0.437	1.12 (0.45–2.79)	0.810	0.54 (0.25–1.16)	0.114	1.04 (0.43–2.54)	0.926
Diagnosis of Depression								
No	1.00 (ref)		1.00 (ref)		1.00 (ref)		1.00 (ref)	
Yes	0.76 (0.48–1.22)	0.256	1.19 (0.57–2.48)	0.650	0.71 (0.38–1.33)	0.281	0.71 (0.27–1.88)	0.495

*High education means university studies or secondary school; Low education: up to primary studies.

^†^Multivariable models were adjusted by: age, smoking, alcohol consumption, body-mass index, hypertension, type 2 diabetes, dyslipidemia and family history of premature coronary heart disease, and type Mediterranean diet intervention.

^‡^Global test of proportional-hazard assumption (p-value based on the scaled Schoenfeld residuals) men: Composite primary cardiovascular event model (0.172); Myocardial infarction model (0.723); Stroke model (0.260); Cardiovascular death model (0.369); women: Global test of proportional-hazard assumption (p-value based on the scaled Schoenfeld residuals) women: Composite primary cardiovascular event model (0.553); Myocardial infarction model (0.320); Stroke model (0.870); Cardiovascular death model (0.458).

A Mediterranean diet with EVOO or nuts showed a protective combined cardiovascular effect. Current and former smokers had a higher risk of suffering cardiovascular events. Hypertensive individuals tended to have a greater risk of experiencing a cardiovascular event. Diabetics presented more risk of stroke (statistical significance at the limit point: p-value: 0.052). No higher risk for participants with dyslipidemia and family history of premature CHD was found (Table 4).

**Table 4:** **Adjusted hazard ratios for cardiovascular outcomes within all covariables include in the final multivariable models**

	Composite primary cardiovascular event		Myocardial infarction		Stroke		Cardiovascular death	
	HR (95% CI) ^†‡^	p-value	HR (95% CI) ^†‡^	p-value	HR (95% CI) ^†‡^	p-value	HR (95% CI) ^†‡^	p-value
Age (years)	1.08 (1.06–1.10)	<0.001	1.04 (1.00–1.07)	0.034	1.09 (1.05–1.12)	<0.001	1.15 (1.11–1.19)	<0.001
Type of Mediterranean diet								
With extra-Virgin Olive oil	1.00 (ref)		1.00 (ref)		1.00 (ref)		1.00 (ref)	
With mixed nuts	0.97 (0.74–1.35)	0.984	0.98 (0.60–1.60)	0.938	0.76 (0.49–1.22)	0.277	1.37 (0.80–2.34)	0.255
Low fat diet	1.42 (1.07–1.88)	0.014	1.35 (0.84–2.15)	0.205	1.44 (0.98–2.12)	0.062	1.38 (0.80–2.38)	0.242
High alcohol consumption	0.85 (0.63–1.15)	0.292	0.59 (0.35–1.00)	0.052	1.17 (0.77–1.77)	0.472	0.98 (0.58–1.66)	0.949
Smoking status								
Never smoked	1.00 (ref)		1.00 (ref)		1.00 (ref)		1.00 (ref)	
Former smoker	1.45 (1–04–2.03)	0.031	1.47 (0.84–2.56)	0.176	1.14 (0.69–1.86)	0.615	2.27 (1.22- 4.24)	0.010
Current-smoker	1.94 (1–33–2.84)	0.001	2.18 (1.20–3.97)	0.011	1.46 (0.82–2.58)	0.197	2.51 (1.20–5.22)	0.014
Body-mass index (Kg/m^2^)	1.01 (0.97–1.04)	0.661	1.01 (0.96–1.07)	0.697	1.00 (0.95–1.04)	0.875	1.03 (0.96–1.09)	0.421
Hypertension	1.79 (1.06–3.02)	0.030	1.53 (0.70–3.32)	0.284	2.19 (0.96–4.99)	0.063	2.22 (0.69–7.08)	0.179
Type 2 diabetes	1.30 (0.98–1.74)	0.071	1.23 (0.78–1.96)	0.374	1.53 (1.00–2.35)	0.052	1.21 (0.71–2.05)	0.477
Dyslipidaemia	0.91 (0.70–1.18)	0.468	0.82 (0.54–1.26)	0.376	0.94 (0.64–1.38)	0.762	0.89 (0.55–1.44)	0.630
Family history of premature CHD^§^	1.24 (0.92–1.68)	0.155	1.56 (0.98–2.48)	0.060	1.25 (0.82–1.91)	0.304	0.70 (0.34–1.40)	0.313

^†^All Hazard Ratios are adjusted by sex and psychosocial factors (educational level, depression and social support in the household).

^‡^Global test of proportional-hazard assumption (p-value based on the scaled Schoenfeld residuals): Composite primary cardiovascular event model (0.471); Myocardial infarction model (0.758); Stroke model (0.303); Cardiovascular death model (0.107).

^§^CHD denotes coronary heart disease.

## Discussion

In our study it was observed that an adult population at high cardiovascular risk, with low education level, had an increased risk of stroke.

Educational level was employed as a measure to evaluate socioeconomic position (SP) because it tends to remain stable throughout life. It is strongly related to the possibility of greater social and material opportunities by influencing future employment and income, and the adoption of healthy lifestyles [17]. As a proxy of SP, educational level has been widely used in a number of cardiovascular research studies [8, 20, 21].

Although, in general, Spain has one of the lowest incidences of cardiovascular diseases, our results linking socioeconomic level and stroke concur with findings from other studies performed in Anglo-Saxon and Scandinavian countries [21–23].

Classic stroke risk factors and harmful lifestyles are likely to be more prevalent among socioeconomically deprived groups [6, 22]. In some studies, the socioeconomic gradient observed in the relationship between SP and stroke was partially mediated by traditional risk and psychological factors [24, 25]. In our population, however, the association between stroke and educational level was independent of the cardiovascular risk factor profile and unhealthy lifestyle, as has previously been observed [26].

CVD mortality is decling in Spain yet we observed a high prevalence of poor management of traditional risk factors, and a low rate of proper treatment for the population at risk. Findings that are in agreement with studies carried out on the population attended by the Health System in Catalunya, a region in Spain [27, 28].

Strong primary healthcare models play a key role in reducing socioeconomic health inequalities [29]. The Spanish system, for instance is both free and universal with GPs acting as gatekeepers to specialist care. Yet we are of the opinion that, in contrast to other countries where health expenditure has been found to be inversely associated with stroke incidence [30, 31], inequality regarding accessibility to health services had a negligible effect on CVD incidence in our population. Indeed, in a previous study we found no differences in the preventive treatment received according to educational level which could justify social inequalities in the incidence of CVD [32].

The fact that the other psychosocial factors considered, such as depression and low social support, were not found to be related to CVD remains to be elucidated. Through the understanding of social disparities in stroke it would be possible to more effectively address social and clinical actions for cardiovascular prevention in the most disadvantaged social groups.

The Spanish population tends to use the health system quite often, as a result, the probability of being treated for comorbid conditions such as depression is high and the effect of this condition on stroke incidence could be reduced by proper treatment. The number of people living alone among our participants was low. It is possible that a longer follow-up or a larger sample might demonstrate some relationship between living alone and CVD incidence. It should also be taken into account that in the Mediterranean countries, families still play an important role in the care of elderly people; therefore, the effect of living alone could be lower than in other countries.

### Strengths and limitations

Unfortunately, no other socioeconomic indicators were available to carry out a sensitivity analysis. Educational level has, however, been found to be a reliable indication of socioeconomic position [17]. With respect to evaluating the effect of social support on cardiovascular outcomes, we took into consideration the number of people living in the home as household size has proven to be a valid proxy [18]. We were, therefore, unable to assess this effect on institutionalized patients or those lacking autonomy. Moreover, since history of depression was self-reported it is possible that the real proportion of depressive patients was under-registered.

Although there are peculiarities in the pathogenesis of hemorrhagic and ischemic stroke, their prevention and management are quite similar, and previous studies have found a relationship between low SP, measured by a deprivation index, for both types. We could not differentiate between hemorrhagic and ischemic stroke because we only had aggregated data [33]. It could be useful in the future to carry out studies to demonstrate whether differences between ischemic and hemorrhagic stroke are due to socioeconomic status.

We are aware that, since several end points have been considered in the analysis and, multiple comparisons among different subgroups of participants may increase type I error. Nevertheless, since our study specifically tested the relationship between psychosocial determinants and cardiovascular events we did not carry out multiple analyses other than those needed to answer the main question.

## Conclusions

In a population at high cardiovascular risk, the incidence of stroke was higher in those with lower educational level. History of depression and low social support were not associated with CVD incidence.

## Abbreviations

CVD: Cardiovascular diseases
SD: Standard deviation
HR: Hazard ratios
CI: Confidence interval
SES: Socioeconomic status
GPs: General practitioners
BMI: Body mass index
CHD: Coronary heart disease
ACE: Angiotensin-converting-enzyme
EVOO: Extra-virgin olive oil.
